# CD80 and CD86 Costimulatory Molecules Differentially Regulate OT-II CD4^**+**^ T Lymphocyte Proliferation and Cytokine Response in Cocultures with Antigen-Presenting Cells Derived from Pregnant and Pseudopregnant Mice

**DOI:** 10.1155/2014/769239

**Published:** 2014-03-19

**Authors:** Tomasz Maj, Anna Slawek, Anna Chelmonska-Soyta

**Affiliations:** ^1^Laboratory of Reproductive Immunology, Ludwik Hirszfeld Institute of Immunology and Experimental Therapy, Polish Academy of Sciences, Weigla 12 Street, 53-114 Wroclaw, Poland; ^2^Faculty of Veterinary Medicine, Wroclaw University of Environmental and Life Sciences, Norwida 31 Street, 50-375 Wroclaw, Poland

## Abstract

Immune phenomena during the preimplantation period of pregnancy are poorly understood. The aim of our study was to assess the capacity for antigen presentation of splenic antigen-presenting cells (APCs) derived from pregnant and pseudopregnant mice in *in vitro* conditions. Therefore, sorted CD11c^+^ dendritic cells and macrophages F4/80^+^ and CD11b^+^ presenting ovalbumin (OVA) were cocultured with CD4^+^ T cells derived from OT-II mice's (C57BL6/J-Tg(TcraTcrb)1100Mjb/J) spleen. After 132 hours of cell culture, proliferation of lymphocytes (ELISA-BrdU), activation of these cells (flow cytometry), cytokine profile (ELISA), and influence of costimulatory molecules blocking on these parameters were measured. We did not detect any differences in regulation of Th1/Th2 cytokine balance. CD86 seems to be the main costimulatory molecule involved in the proliferation response but CD80 is the main costimulatory molecule influencing cytokine secretion in pregnant mice. In conclusion, this study showed that CD80 and CD86 costimulatory molecules regulate OT-II CD4^+^ T lymphocyte proliferation and cytokine response in cocultures with antigen-presenting cells derived from pregnant and pseudopregnant mice. The implications of these changes still remain unclear.

## 1. Introduction

It is well established that tolerance of maternal immune system is decisive for pregnancy success. The tolerogenic maternal immune response against paternal alloantigens not only is achieved in the presence of a fetus, but also may be a consequence of several overlapping physiological mechanisms. Most of the immune responses associated with pregnancy have been described in the context of mid-gestation, while the shaping of immune mechanisms in early pregnancy, especially within the preimplantation period, is still poorly understood. Three events inducing immune tolerance against a semiallogeneic conceptus may play a crucial role in the preimplantation period of pregnancy: (i) the influence of sex hormones in the sex cycle [1–6]; (ii) the presence of an oocyte or embryo [7–10]; (iii) and the presence of semen in the female reproductive tract [11–18]. We paid attention to this period of pregnancy because it seems to be significant for establishment of peripheral tolerance to fetal antigens without impairment of the capability of effective anti-infectious defense. Therefore, potent activity of antigen-presenting cells (APCs) may be crucial for these events. In a previous study we found that mating changed the level of costimulatory molecules CD40, CD80, and CD86 and MHC class II on splenic APCs (CD11c^+^, F4/80^+^, CD11b^low^, and CD11b^high^) before implantation [19].

In opposition to local response, peripheral awareness of early pregnancy may be decisive for a generation of split tolerance, which is believed to operate during pregnancy of placental mammals [20]. Differential expression of costimulatory molecules on spleen APCs of mated v. pseudopregnant mice was observed by us mainly at day 3.5 after conception. Moreover, we observed that the costimulatory potential of F4/80^+^ macrophages measured by the expression level of costimulatory molecules seemed to be higher in comparison with other populations of APCs studied by us. In an* in vivo* experiment, where blocking antibodies against costimulatory molecules were given i.p. at day 3.5 after mating, cytokine expression was modulated after administration of anti-CD40, anti-CD80, and anti-CD86 at day 10.5 [19]. Administration of anti-CD40 (stimulating antibody) and anti-CD86 (blocking antibody) decreased the possibility of pregnancy, whereas blocking the CD40 molecule led to an increase of Treg lymphocyte concentrations. We hypothesize that the changes in the levels of CD80 and CD86 during preimplantation period of pregnancy have functional meaning and are directly connected with regulation of T cell response. Therefore, we evaluated antigen presentation potency of splenic APCs isolated from mice in the preimplantation stage of pregnancy. For this aim, sorted dendritic cells CD11c^+^ and F4/80^+^ and CD11b^+^ macrophages loaded with ovalbumin (OVA) were cultured with CD4^+^ T cells derived from OT-II mice's (C57BL6/J-Tg(TcraTcrb)1100Mjb/J) spleen. We found that proliferation of CD4^+^ T lymphocytes depends entirely on CD86 availability both in pregnant and pseudopregnant mice; however, cytokine production in pregnancy is mainly regulated by the CD80 costimulatory molecule.

## 2. Materials and Methods

### 2.1. Animals

Adult (8-week-old) female C57BL/6J, male DBA/2J, and male Balb/c strains of mice were purchased from the Experimental Medicine Center, Medical University of Bialystok (Poland). OT-II mice were purchased from Charles River Laboratories (France). The animals were housed in a constant light-to-dark ratio of 12 : 12 hours under specific pathogen free (SPF) conditions. All described procedures were approved by the Local Ethics Committee of the Institute of Immunology and Experimental Therapy in Wroclaw (permission number 4/2009).

### 2.2. Mating and Induction of Pseudopregnancy

The stage of the estrous cycle was determined every day (8:00–10:00 AM) by cytology of vaginal smears. The smears were stained with a Cytocolor kit (Merck, Germany), according to the manufacturer's instructions. Females in estrus were mated with the male Balb/c or DBA/2J mice. The female-to-male ratio was 1 : 1. The act of mating was confirmed by the presence of a vaginal plug next morning (considered as day 0.5). Because the presence of the vaginal plug does not verify pregnancy, these animals are described as mated. Pseudopregnancy was induced by mechanical stimulation of the uterine cervix when the females were in estrus. Pseudopregnancy was confirmed by analysis of vaginal smears in the morning after stimulation and in consecutive days before killing. This control has an important advantage over the control consisting of cyclic nonpregnant mice because the pseudopregnant mice have the same endocrine background as pregnant females. Animals displaying the continuous microscopic image of metestrus were recognized as pseudopregnant. At 3.5 days after mating, mice were euthanized by cervical dislocation.

### 2.3. Isolation of Spleen Cells

Freshly acquired spleens were weighed and immediately pushed through a 40 *μ*m sieve to obtain a single cell suspension. The cells were incubated for 1 min in 0.84% ammonium chloride solution for erythrocytes lysis. Next, leucocytes suspensions were centrifuged (400 g, 5 min) and supernatants were discarded. To remove debris, the cells were washed twice in Dulbecco's PBS [21].

### 2.4. Magnetic Cell Sorting

CD11c^+^, CD11b^+^, and F4/80^+^ were isolated from a suspension of total spleen cells in PBS supplemented with 2 mM EDTA (Sigma-Aldrich, Poznan, Poland) and 0.5% BSA (Sigma-Aldrich) according to the producer's instructions. We used a magnet, columns, and magnetically labeled antibodies from Miltenyi Biotec. In the case of F4/80^+^ cells we performed indirect staining with anti-F4/80 FITC (eBioscience, Vienna, Austria) and anti-FITC magnetic beads, whereas CD11c^+^ and CD11b^+^ cells were labeled directly. CD4^+^ T cells were isolated with a kit for negative selection (depletion of non-CD4^+^ cells). In the case of splenic APCs, we sorted CD11c^+^ at the beginning, next F4/80^+^ cells, and CD11b^+^ cells at the end. In all cases the purity of sorted cells was 85% or higher, as determined by flow cytometry.

### 2.5. T Cells/APC Cocultures

Sorted CD11c^+^, F4/80^+^, and CD11b^+^ cells were suspended in RPMI 1640 (Sigma-Aldrich), 10% fetal calf serum (FCS, Invitrogen), and L-glutamine-penicillin-streptomycin stock from Sigma-Aldrich 1 : 100 and treated with mitomycin C (Santa Cruz Biotech.) for 15 min and at 37°C. After triple washing in PBS, the cells were resuspended in complete RPMI 1640 supplemented with 10 *μ*M OVA (Sigma-Aldrich). After counting, the cells were diluted to a concentration of 6 × 10^5^ and 50 *μ*L of suspension was added to a 96-well U-bottom culture plate (Becton Dickinson). The cells were treated with suppressive Armenian hamster anti-mouse CD80 (clone 16-10A1) and suppressive rat anti-mouse CD86 antibody (clone PO3.1; all functional grade antibodies were purchased from eBioscience). Control cells were treated with appropriate isotype controls: rat IgG2a or Armenian hamster IgG (eBioscience, functional grade immunoglobulins). After 30 min treatment with antibodies we added OT-II CD4^+^ T cells (6 × 10^3^ cells per well). The total volume for each well was 100 *μ*L and final concentration of each antibody was 5 *μ*g/mL. The cells were incubated for 6 days, in 5% CO_2_ and at 37°C. APC : T cell ratio was 5 : 1. These cells were cocultured for 132 hours.

### 2.6. BrdU Proliferation Assay

Certain cocultures were destined for proliferation assay with the colorimetric BrdU proliferation ELISA kit (Roche). Briefly, the cells were treated with bromodeoxyuridine (BrdU) 12 hours before the end of incubation. After centrifugation of plates, the pellets were dried for 15 min with a hair dryer and fixed/denatured for 60 min at room temperature (RT), according to the producer's instructions. Next, the plates were blocked for 30 min with 2% BSA solution and BrdU was detected by direct staining with anti-BrdU-POD antibody (90 min, RT). After triple washing, an enzymatic reaction with TMB as the substrate was carried out for 7 min and impeded with H_2_SO_4_. Absorbance at wavelength *λ* = 450 nm was measured within 15 min after H_2_SO_4_ addition. Since the procedure was performed on distinct plates and days, isotype control treated cells were considered as a reference measurement and the results of costimulatory molecule blockade are depicted as a percentage of the reference value.

### 2.7. Cytokines ELISA

Supernatants from cocultures were stored at −80°C after collecting. IFN-gamma, IL-2, IL-4, IL-10, IL-12, and TGF-beta were measured using murine Ready-SET-Go kits from eBioscience. Briefly, Costar 96-well ELISA plates were coated with specific antibodies overnight (4°C) and blocked for 1 hour, RT. Next, supernatants diluted 1 : 10 and standard concentration of cytokines (100 *μ*L/well) were incubated overnight at 4°C, washed three times, and incubated with biotinylated specific detection antibodies (60 min, RT). After further triple washing, we added horseradish peroxidase-conjugated avidin and incubated the plates for 30 min, RT. Washed plates were next incubated for 10 min in the dark at RT with TMB substrate and inhibited with 50 *μ*L of 1 M H_2_SO_4_.* A*_450_ was measured on a spectrophotometer within 15 min from the protocol endpoint.

### 2.8. Flow Cytometry

All antibodies and isotype controls for cell labeling were purchased from eBioscience (USA) and were diluted in PBS with 2% normal mouse serum (Sigma-Aldrich). After 132-hour coculture incubation, the cells were stained in a total volume of 50 *μ*L with anti-CD4-FITC (clone RM4-5, final concentration 1.2 *μ*g/mL) and anti-CD25-PE (clone PC61.5, 0.24 *μ*g/mL) or isotype controls which were used at the same concentration as specific antibodies. After 45 min of incubation at 4°C, the cells were washed twice in PBS and then fixed in 1% buffered formaldehyde. The samples were acquired with a FACSCalibur II Cytometer (Becton, Dickinson and Company). We performed FACS analysis to determine the ratio of total number of activated (CD4^+^CD25^+^) T cells to nonactivated (CD4^+^CD25^−^) cells.

### 2.9. Statistics

All statistical calculations were performed in Statistica 7 (StatSoft). The shape of data distribution was assessed with the Shapiro test and analysis of quantile-normal plots. Homoscedasticity was tested with Levene's test. Student's *t-*test (parametric) or Wilcoxon (nonparametric) test was performed on the basis of data distribution shape.

## 3. Results

### 3.1. Proliferation of OT-II CD4^+^ T Cells Depends on Availability of CD86

**Figure 1:**
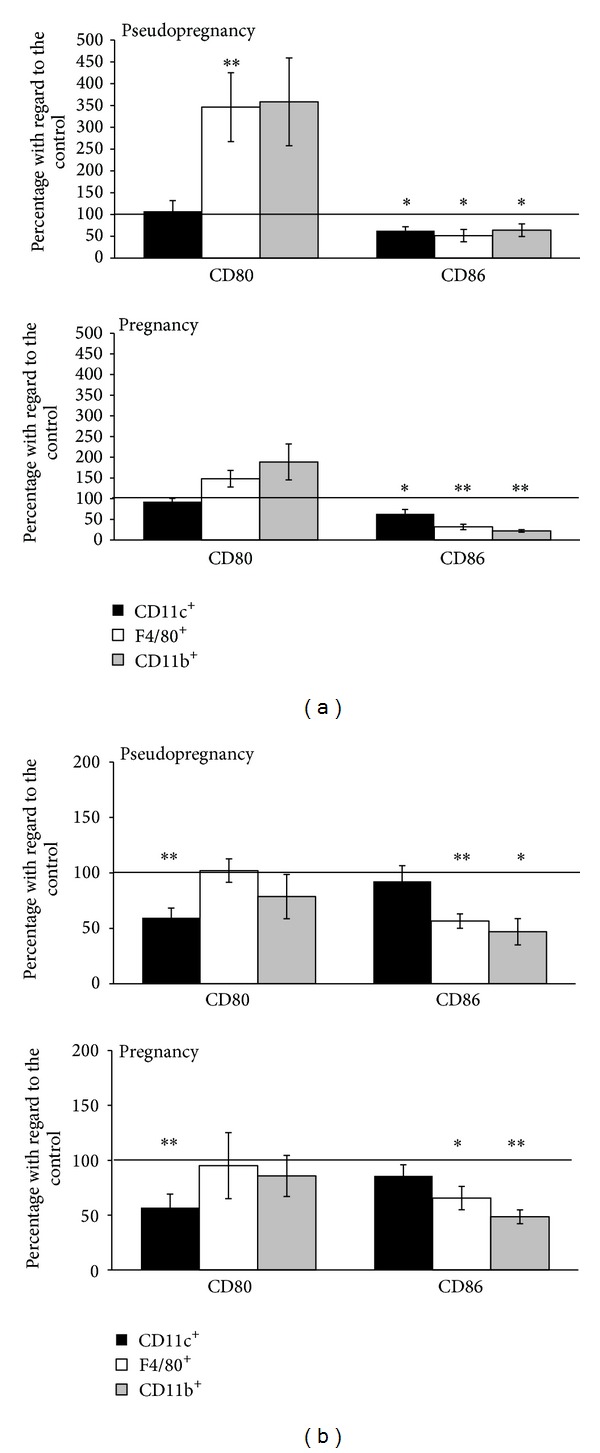
Proliferation and activation of OVA-specific T cells cocultured with splenic APCs isolated from mated or pseudopregnant female mice after blockade of CD80 and CD86. (a) Proliferation estimated by BrdU incorporation and ELISA. Data were presented as percentages of absorbance for blocked molecules in comparison with isotype control (mean ± SEM). (b) Activation of T cells was expressed as the ratio of total number of CD4^+^CD25^+^ cells to the total number of CD4^+^CD25^−^ cells. Data were presented as percentages of CD4^+^CD25^+^/CD4^+^CD25^−^ for blocked molecules in comparison with isotype control (mean ± SEM). *N* = 10 animals per group; Student's *t*-test (parametric) or Wilcoxon (nonparametric) test, *P < 0.05; **P < 0.01.

**Figure 2:**
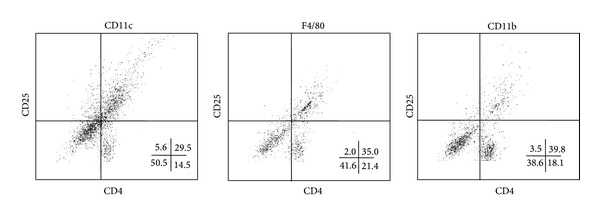
Representative dot-plots of flow cytometric analysis of an expression of CD25 molecules on OT-II CD4^+^ lymphocytes after coincubation with CD11c^+^, F4/80^+^, and CD11b^+^ spleen cells isolated from pregnant mice.

To assess the involvement of CD80 and CD86 molecules in activation of T cells, we enriched murine splenic APCs populations: macrophages (F4/80^+^ or CD11b^+^ cells) and dendritic cells (CD11c^+^ cells). The cells were obtained from both pseudopregnant mice (control) and mated animals (experimental group). Next, the APCs were loaded with OVA and exposed to OVA-specific OT-II CD4^+^ T cells. Additionally, the cocultures were treated with anti-CD80 and anti-CD86 blocking antibodies or relevant control immunoglobulins. After 6 days we performed the test for proliferation and expression of T cell activation marker CD25. The proliferation test revealed that in most cases treatment of costimulatory molecules with specific antibodies led to similar effects in mated and pseudopregnant mice. The blockade of CD80 significantly increased the proliferation of T cells only in the presence of F4/80^+^ cells obtained from pseudopregnant animals (*P* = 0.002; Figure 1(a)). Although the capability of CD11b^+^ cells to elicit T cells proliferation seemed to be similar, this observation was not significant due to higher variance. Insignificant increases of proliferation were also observed after anti-CD80 treatment in the same populations of APCs in pregnancy. The CD80 molecule blockade seemed to have completely no effect on OT-II T cell proliferation in culture with dendritic cells. On the other hand, blockade of CD86 led to decreased proliferation ratio of these cells in cultures with all types of studied APCs both in pregnancy (*P* = 0.002 for CD11c^+^ cells; *P* = 0.005 for F4/80^+^ cells; *P* = 0.007 for CD11b^+^ cocultures) and pseudopregnancy (*P* = 0.01, *P* = 0.02, and *P* = 0.04, resp.) (Figure 1(a)). Additionally, we performed FACS analysis to determine the ratio of total number of activated (CD25^+^) and nonactivated (CD25^−^) CD4^+^ T cells. The representative flow cytometry dot-plots are shown in Figure 2. The changes in the ratio of CD25^+^/CD25^−^ T cell number were convergent with the proliferation test in the case of CD86 blockade on F4/80^+^ (*P* = 0.01 in pregnancy; *P* = 0.008 in pseudopregnancy) and CD11b^+^ macrophages (*P* = 0.002 and *P* = 0.01, resp.). However, treatment of CD11c^+^/T cell cocultures with anti-CD80 led to a decrease in CD25^+^/CD25^−^ ratio (*P* = 0.006 for pregnancy and *P* = 0.008 for pseudopregnancy) (Figure 1(b)). Notably, the proliferative response was not affected under these conditions (Figure 1(a)).

### 3.2. Cytokine Profiles Differ in the Presence of Anticostimulatory Molecules* In Vitro*

**Figure 3:**
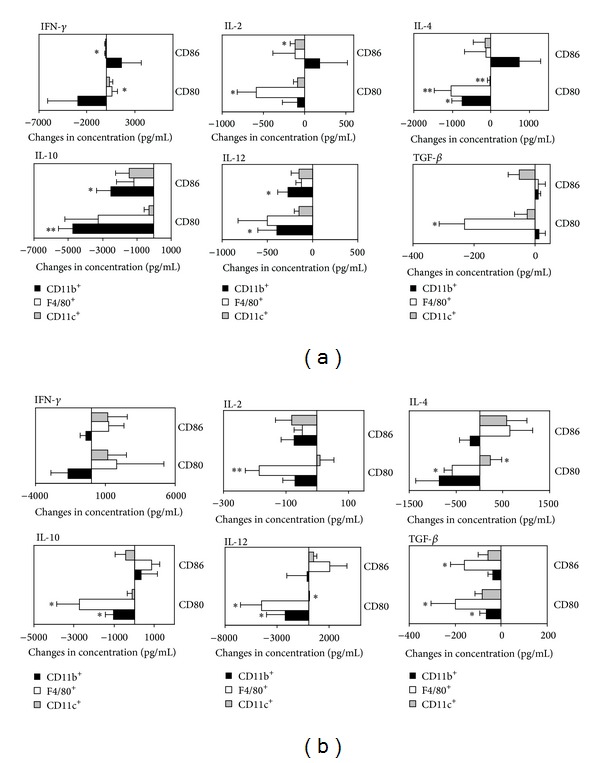
Changes in cytokine profile detected in cocultures of OVA-specific T cells with splenic APCs isolated from pseudopregnant (a) or mated (b) female mice after blockade of costimulatory molecules CD80 and CD86. Data were presented as increase/decrease of concentration in comparison to appropriate isotype control-treated cultures (mean ± SEM). *N* = 10 samples per group. Student's *t*-test (parametric) or Wilcoxon (nonparametric) test, *P < 0.05; **P < 0.01.

Next, in supernatants from the above-mentioned cocultures we assessed the level of IFN-*γ*, IL-2, IL-4, IL-10, IL-12, and TGF-*β* by ELISA (Figure 3). The concentration of IFN-*γ* was significantly changed only in cocultures of OVA-specific T cells with F4/80^+^ macrophages (*P* = 0.02 for both anti-CD80 and anti-CD86 treatment; Figure 3(a)) isolated from pseudopregnant but not mated animals. IL-2 level was downregulated after blockage of CD80 on F4/80^+^ cells isolated from both mated (*P* = 0.003; Figure 3(b)) and pseudopregnant (*P* = 0.028; Figure 3(a)) mice. In pseudopregnancy we also observed a decrease of the IL-2 level in cultures containing CD86-blocked CD11b^+^ cells (*P* = 0.02; Figure 3(a)). IL-4 concentration was affected only in anti-CD80-treated cells. Blockade of CD80 in B6 mice led to a decrease of IL-4 concentration in cocultures with all studied APCs populations (*P* = 0.02 for CD11c^+^ cells, *P* = 0.007 for F4/80^+^ macrophages, and *P* = 0.009 for CD11b^+^ cells; Figure 3(a)), but in mated females we observed a significant decrease in the presence of F4/80^+^ (*P* = 0.028; Figure 3(b)) and—in contrast to pseudopregnant mice—an increase in cocultures with CD11b^+^ cells (*P* = 0.046; Figure 3(b)). We observed a decrease in IL-10 concentration in the presence of CD11c^+^ cells in pseudopregnant and pregnant mice (*P* = 0.005 and *P* = 0.04, resp.). The difference between pseudopregnant and pregnant mice was related to a decrease of IL-10 concentration in cocultures with CD80-blocked F4/80^+^ cells (*P* = 0.01; Figure 3(b)) and a decrease of this cytokine level in cocultures with CD86-blocked CD11c^+^ cells (*P* = 0.04; Figure 3(a)). In the case of IL-12, the concentration was downregulated in cocultures with CD11c^+^ cells of B6 animals treated with anti-CD80 Ab (*P* = 0.046) and anti-CD86 Ab (*P* = 0.04) (Figure 1(a)). IL-12 level was affected by CD80 blockade of all studied APC populations isolated from pregnant mice (*P* = 0.018 for CD11c^+^ cells; *P* = 0.018 for F4/80^+^ cells; *P* = 0.028 for CD11b^+^ cocultures; Figure 3(b)). TGF-*β* concentration was decreased after blockade of stimulation of T cells (anti-CD80 Ab) with F4/80^+^ cells isolated from both control (*P* = 0.01; Figure 3(a)) and experimental mice (*P* = 0.04; Figure 3(b)). However, in mated but not pseudopregnant animals anti-CD80 Ab treatment led to a decrease in concentration in the case of CD11c^+^ cells (*P* = 0.03). Addition of anti-CD86 Ab to cocultures prompted a decrease in the level in the case of F4/80^+^ (*P* = 0.036) (Figure 3(b)). Cumulatively, these results led to the observation that CD80 is the main costimulatory molecule influencing secretion of cytokines in pregnant mice (Table 1).

## 4. Discussion

Our research on CD4^+^ T lymphocytes and the cytokine secretion profile in* in vivo* [19] and* in vitro* conditions highlights the distinct role of CD80 and CD86 molecules. Data concerning other experimental models, such as human normal pregnancy and miscarriage, experimental autoimmune encephalomyelitis (EAE), or a murine model of leishmaniasis, also indicate differential action of these costimulatory molecules. It is obvious that these costimulatory molecules take part in the regulation of cytokine production. However, each of them may be differentially engaged in the regulation of cytokines synthesis. For instance, the level of mRNA expression for CD86, but not CD80, is positively correlated with the level of Th1 cytokines during human miscarriage [22]. In a model of EAE a blockade of CD80 or CD86 has different effects: blockade of CD80 interactions with its ligands leads to production of Th2 cytokines and cessation of disease symptoms, while CD86 blocking has adverse effects [23]. In a murine model of leishmaniasis a blockade of CD86 results in inhibition of Th2 cytokine secretion and enhancement of the Th1-type immune response, while a blockade of CD80 exerts the opposite effect [24]. On the other hand, in* in vitro *experiments Th1/Th2 mechanisms are not strictly connected with the presence of CD80 or CD86. Data from coculture experiments of antigen-specific T cells with APCs isolated from CD80 KO or CD86 KO mice indicate that CD86 and, to a lesser extent, CD80 are involved in regulation of both IFN-*γ* and IL-4 production, but without preferential support of a Th1 or Th2 response [25]. Similarities in CD80 and CD86 action under the scope of Th1/Th2 balance were demonstrated in other experimental models, for example, in human PBMCs or murine CD4^+^ T lymphocytes cultured in* in vitro* conditions [26–28]. Additionally, the outcome of cytokine production is dependent on other factors, such as antigen density and priming/restimulation. For example, if CD4^+^ T cells recognize an antigen with low affinity/avidity, they are committed to Th2 cells; otherwise, they are redirected to Th1 development [29]. Moreover, the role of CD80 and CD86 can be distinct in priming or restimulation, since previous* in vitro* study demonstrated that blockade of CD86 influences the production of IL-4, but CD80 blockade has an effect only after restimulation and is related to both IL-4 and IFN-*γ* [30]. In the context of pregnancy, the blockade of CD80 and CD86 molecules in early stages of gestation abortion-prone mice (CBA/JxDBA/2) increased production of Th2 cytokines and frequency of Treg cells and decreased the fetal resorption [31, 32].

In our* in vitro *experiments we observed that cytokine production from CD4^+^ OT-II T cells cocultured with splenic APCs after blockade of CD80 and CD86 is not specifically related to Th1 or Th2 profile (Table 1). However, we can draw a few other conclusions. Firstly, the influence of blockade of costimulatory molecules with specific Ab is cell-type dependent. From studied populations of splenic APCs, blocking of costimulatory molecules affected mainly F4/80^+^ cells, whereas CD11c^+^ and CD11b^+^ cells were targeted to a lesser extent. In a previous study we observed that at embryonic day 3.5 all studied costimulatory molecules (CD40, CD80, and CD86) were upregulated on the surface of F4/80^+^ cells, in contrast to the population of CD11c^+^ and CD11b^+^, where the observed increased expression of these molecules was not even [19]. Thus, we can speculate that F4/80^+^ macrophages may be the main cell population of murine splenic APCs affected during the preimplantation period of pregnancy and these cells are primarily linked with the regulation of the immune response. Secondly, in pregnancy most changes were induced by blockade of CD80 and resulted in diminished secretion of both Th1 and Th2 cytokines. Only TGF-beta production was diminished by a blockade of both costimulatory molecules. However, an increase in cytokines production (IL4 and IL-12) was also observed, but only in the case of CD11b^+^ cells. In pseudopregnancy, both costimulatory molecules were involved in cytokine production regulation. Thirdly, in pregnant mice IFN-*γ* production was not dependent on availability of both costimulatory molecules. Additionally, the proliferation of T cells was decreased after blockade of CD86 both in pregnant and pseudopregnant mice while CD80 blockade had no effect on proliferative response except for F4/80^+^ macrophages as stimulatory cells in the group of pseudopregnant mice, where they led to an increase of proliferation. Moreover, the changes in ratio of CD25^+^ to CD25^−^ T cells were convergent with the results of BrdU assay; CD86 blockade on F4/80^+^ and CD11b^+^ macrophages suppressed both proliferation and generation of activated T cells. Since this effect is not visible in cocultures of T cells with DCs, this observation suggests that CD86 may regulate activation of T cells during their interaction with macrophages. On the other hand, the effect of anti-CD80 treatment limited frequency of activated T cells only in the presence of DCs, but not macrophages. In this case, however, the intensity of proliferation was not affected, while the concentrations of studied cytokines were clearly affected by CD80 blockade. Thus, CD86 seems to be the main costimulatory molecule involved in the proliferation response in both pregnant and pseudopregnant mice, whereas CD80 is engaged in stimulation of cytokine production, especially in the group of pregnant mice (Table 1). Nevertheless, in our previous paper we showed that splenic APCs during preimplantation period of pregnancy clearly upregulate CD86, while CD80 expression was changed to a lesser extent [19]. Therefore, our current observation indicating that blockade of CD80 decreases the cytokines synthesis can be irrelevant in the* in vivo* models and require more detailed analysis. Still, the effect of CD86 blockade is consistent with our former study. We postulate that regulation of CD86 expression on macrophages may be an important mechanism tuning tolerant immune response during early stages of pregnancy.

## 5. Conclusions

In summary, this study showed that CD80 and CD86 costimulatory molecules differentially regulate OT-II CD4^+^ T lymphocyte proliferation and cytokine response in cocultures with antigen-presenting cells derived from pregnant and pseudopregnant mice. Proliferation of T cells depends on the availability of CD86 molecules, but CD80 molecules are involved in the regulation of cytokines secretion in pregnant mice. It seems that CD80 phenotype does not promote cytokine production in pregnancy.

## Figures and Tables

**Table 1:** Summary of influence of blockade of CD80 and CD86 costimulatory molecules on proliferation of OT-II CD4^+^ T lymphocytes and cytokine concentration in pseudopregnant and pregnant mice.

	Pseudopregnancy						Pregnancy					
	CD11c^+^		F4/80^+^		CD11b^+^		CD11c^+^		F4/80^+^		CD11b^+^	
	80	86	80	86	80	86	80	86	80	86	80	86
IFN-g			↑	↓								
IL-2			↓			↓			↓			
IL-4	↓		↓		↓				↓		↑	
IL-10	↓	↓					↓		↓			
IL-12	↓	↓					↓		↓		↑	
TGF-b			↓				↓		↓	↓		
BrdU		↓	↑	↓		↓		↓		↓		↓

↑: increased proliferation or cytokine concentration, ↓: decreased proliferation or cytokine concentration.
